# Ezetimibe in Combination With a Statin Does Not Reduce All-Cause Mortality

**DOI:** 10.4021/jocmr1371w

**Published:** 2013-06-21

**Authors:** Akshar Y. Patel, Jayasree Pillarisetti, Joshua Marr, James L. Vacek

**Affiliations:** a Department of Medicine, Washington University School of Medicine, St. Louis, MO, USA; b Department of Internal Medicine, University of Kansas School of Medicine and Medical Center, Kansas City, KS, USA

**Keywords:** Ezetimibe, LDL, HDL, Mortality

## Abstract

**Background:**

Although the ezetimibe-statin combination has been shown to reduce LDL cholesterol by 12% compared to a statin alone, its effect on hard clinical endpoints such as mortality is less certain. Prior trials evaluated this combination in highly select population groups, but impact on all- cause mortality in the general population has not been reported.

**Methods:**

A total of 3,827 subjects who were prescribed either a statin (group 1) or the combination of statin with ezetimibe (group 2) between January 1st, 2005 and January 1st, 2008 were studied. Socio-demographic and clinical variables and mortality records were analyzed. Univariate and stepwise multivariate logistic regression analysis was performed to identify the impact of ezetimibe on all-cause mortality, controlling for patient characteristics, selected cardiovascular diseases and risk factors, and medications.

**Results:**

Group 1 (n = 2,909), and group 2 (n = 918) were similar in regards to most demographic variables, 152 patients died from any cause during the study period. There was no difference in all cause mortality between the groups. Hypertension, higher HDL-C and omega-3 fatty acid use were associated with ezetimibe use in this cohort of patients and were considered as covariates in the analysis. Patients on the drug combination did not experience lower mortality after controlling for covariates and other significant risk factors.

**Conclusions:**

No significant mortality benefit was found with the use of ezetimibe in combination with a statin over use of a statin alone. Omega-3 fatty acid use and higher HDL-C demonstrated a substantial survival benefit.

## Introduction

Ezetimibe is a cholesterol lowering medication that works by blocking enteric and biliary absorption of cholesterol, although its exact mechanism of action and affect on various other body systems related to atherosclerosis are under debate [1]. Approved in 2002, Ezetimibe in combination with a statin has been shown to reduce LDL cholesterol ((LDL-C)) 15% more compared to a statin taken alone [2]. Ezetimibe as a monotherapy has been shown to reduce LDL-C by18 % when compared to a placebo [2, 3]. Because the association between LDL-cholesterol reduction and decreased cardiovascular risk has been clearly established, ezetimibe was assumed to possess clinical efficacy and became routinely prescribed [4].

Although the effectiveness of ezetimibe in lowering cholesterol has been demonstrated in several studies, its clinical efficacy has been called into question following the ENHANCE trial (Ezetimibe and Simvastatin in Hypercholesterolemia Enhances Atherosclerosis Regression) which showed that there was no difference in the carotid intima-media thickness in patients taking simvastatin and ezetimibe compared to those on simvastatin alone, despite having reduced overall total cholesterol, LDL-C, triglycerides , and C-reactive protein [5].

Other studies that followed yielded conflicting results with SANDS (Stop Atherosclerosis in Native Diabetics Study) and VYCTOR (Vytorin on Carotid Intima-Media Thickness and Overall Arterial Rigidity) demonstrating a reduction in carotid intima-media thickness. However the ARBITER 6 HALTS trial (Arterial Biology for the Investigation of the Treatment Effects of Reducing Cholesterol 6-HDL and LDL Treatment Strategies in Atherosclerosis) showed no improvement in intima-media thickness with the addition of ezetimibe. Further, the impact of ezetimibe on hard clinical endpoints such as mortality and cardiovascular events has not been proven till date [6-8]. Two studies (SEAS: Simvastatin and Ezetimibe in Aortic Stenosis, and SHARP: Study of Heart and Renal Protection) that demonstrated reduction in major cardiovascular events involved comparison of patients taking a combination of a statin plus ezetimibe versus those taking a placebo alone [9, 10]. The true clinical effect of ezetimibe can only be inferred by using a comparison group that takes a statin alone.

We therefore conducted a retrospective cohort study to compare differences in all-cause mortality between patients taking ezetimibe and a statin, and those on monotherapy with a statin alone.

## Methods

**Figure 1:**
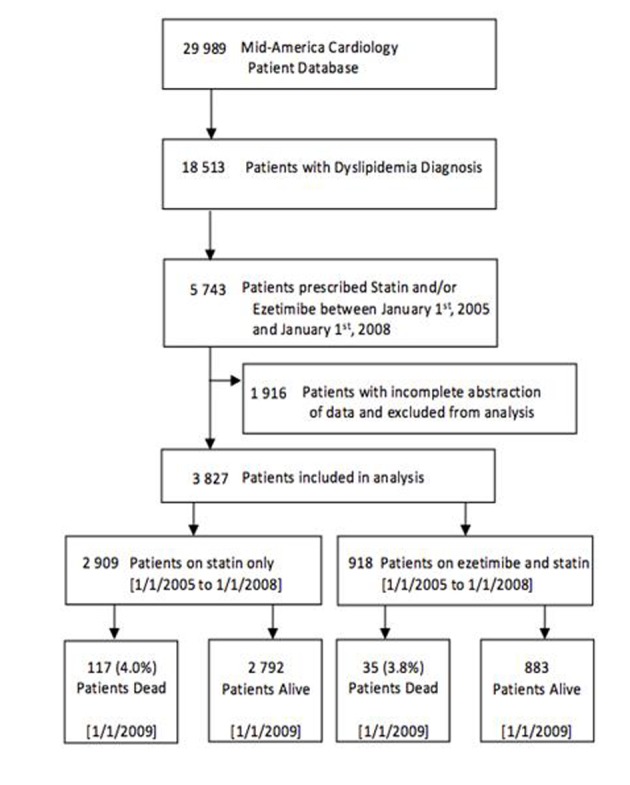
Flowchart of cohort assembly.

A flowchart of cohort assembly can be found in Figure 1. The University of Kansas Hospital Mid-America Cardiology database of electronic patient medical records was used for this study. The study was carried out after obtaining approval from the human subjects committee and waiver of consent for data retrieval.

All social, demographic and clinical variables were obtained from the electronic database. Baseline lipid values of all patients on a statin prior to initiation of ezetimibe were also retrieved.

Study population; Patients who were not diagnosed with dyslipidemia were excluded, and only patients who were prescribed a statin were included. Patients taking combination ezetimibe and a statin (group 1) were compared to those prescribed only a statin (group 2). In order to account for differences in prescribing patterns due to the novelty of ezetimibe, patients who were prescribed either medication before January 1st 2005 were excluded. There was a dramatic fall in ezetimibe prescriptions after articles with negative and inconclusive findings were published, therefore new prescriptions after January 1st, 2008 were also excluded [5].

Follow-up: All patients were followed until January 2009. Patients who were on either medication for at least 1 year and died during this time period were noted.

### Endpoint

The study’s primary endpoint was all cause mortality.

### Statistical analysis

Continuous variables between the groups were compared using unpaired t tests or Wilcoxon two-sample tests depending on whether the variables were normally distributed, and categorical variables were compared using two-sided chi square tests. Characteristics between patients prescribed or not prescribed ezetimibe are compared using the same methods. Odds ratios were calculated using logistic regression and 95% confidence intervals. Logistic regression modeling was done to determine whether measured variables predicted if a patient had greater odds of being prescribed ezetimibe, and stepwise logistic regression was used to determine the optimal model. Odds of mortality were then calculated for each variable using logistic regression. All variables were put into a full model to control for confounding, and stepwise regression was used to determine the optimal model Statistical analysis of the data was performed using SAS version 9.1.3 (SAS Inc, North Carolina, USA). Statistical significance was considered present when P < 0.05.

The authors of this manuscript have certified that they comply with the Principles of Ethical Publishing in the International Journal of Cardiology [11].

## Results

There were some significant differences between the groups (Table 1). The ezetimibe plus statin group was younger (P < 0.001) and had lower HDL-C levels (P = 0.050) with higher incidence of coronary artery disease (P < 0.001) and CABG procedures (P = 0.019) in comparison to the statin only group. Hypertension was more prevalent in the statin-only group (P = 0.004). There was no difference between the total cholesterol (P = 0.785) or LDL (P = 0.116) between the two groups at baseline.

**Table 1:** Characteristics of Ezetimibe Plus Statin Versus Statin Only Patients

Characteristic	Statin and Ezetimibe (n = 918)	Statin only (n = 2,909)	P-value
Gender (Male)	517 (56%)	1,596 (55%)	0.447
Age	62.1 ± 11.5	63.6 ± 12.3	0.001
BMI	31.5 ± 6.9	31.2 ± 7.6	0.413
Weight	203.7 ± 50.8	200.7 ± 51.3	0.119
History of Tobacco use	517 (56%)	1,636 (56%)	0.970
Cholesterol	170.1 ± 62.3	169.6 ± 46.3	0.785
LDL-C	96.2 ± 42.1	96.6 ± 40.3	0.116
HDL-C	47.1 ± 13.3	48.2 ± 14.4	0.050
Hypertension	600 (65%)	2,051 (71%)	0.004
Coronary Artery Disease	405 (44%)	1,027 (35%)	< 0.001
Diabetes	250 (27%)	838 (29%)	0.378
Myocardial Infarction	47 (5%)	160 (6%)	0.738
CABG	60 (7%)	131 (5%)	0.019
PCI	91 (10%)	245 (8%)	0.181
Cerebrovascular Accident	42 (5%)	161 (6%)	0.273
Transient Ischemia Attack	34 (4%)	120 (4%)	0.630
Death	35 (4%)	117 (4%)	0.652
Beta-blocker	605 (66%)	1,838 (63%)	0.145
ACE-I	473 (52%)	1,584 (54%)	0.129
ARB	185 (20%)	547 (19%)	0.361
Omega-3	378 (41%)	935 (32%)	< 0.001
Aspirin	722 (79%)	2,165 (74%)	0.009
Diuretic	411 (45%)	1,319 (45%)	0.790

ACE-I: angiotensin converting enzyme inhibitor; ARB: angiotensin II receptor blocker; BMI: body mass index; CABG: coronary artery bypass graft; HDL-C: high density lipoprotein cholesterol; LDL-C: low density lipoprotein cholesterol; PCI: percutaneous coronary intervention.

Logistic regression was used to determine whether certain characteristics increased the odds of a patient being prescribed ezetimibe (Table 2). Using stepwise regression, three variables significantly changed the odds. Patients with higher HDL levels had lower odds of being on ezetimibe in addition to a statin. Patients diagnosed with hypertension were 19.5% less likely to be on both medications, and those taking omega-3 were 69% more likely to be taking ezetimibe.

**Table 2:** Odds of Being Prescribed Ezetimibe

Characteristic	Unadjusted Odds Ratio	Stepwise Model
HDL-C	0.994 (0.989 - 0.999)	0.992 (0.985 - 1.000)
Hypertension	0.805 (0.705 - 0.919)	0.758 (0.609 - 0.942)
Omega-3 fatty acids	1.689 (1.483 - 1.923)	1.457 (1.178 - 1.802)

HDL-C: high density lipoprotein cholesterol.

The odds of mortality based on the measured demographic variables, cholesterol levels, disease diagnoses, cardiovascular procedures, and cardiovascular medications were each calculated independently using logistic regression (Table 3). No survival advantage was seen with ezetimibe added to a statin (4% in both groups). When controlling for all measured factors, advancing age, a history of smoking, hypertension, a lower HDL level, having diabetes, taking beta-blockers, taking diuretics, and not taking omega-3 fatty acids were all significantly associated with mortality. Ezetimibe dropped out of the final model and was then added back to find a final odds ratio. As could be predicted from looking at Table 1, a patient who takes both ezetimibe and statins was not found to benefit with regard to all cause mortality from a patient taking only statins (OR 1.067, 95% CI: 0.713 - 1.598). Omega-3 fatty acid use (OR 0.554, 95% CI: 0.373 - 0.823) or a higher HDL-C (OR 0.974, 95% CI: 0.959 - 0.989) emerged as significantly protective of all cause mortality.

**Table 3:** Odds of Mortality Based on Cardiovascular Risk Factors

Characteristic	Unadjusted Odds Ratio	Adjusted Odds Ratio	Stepwise Model
Gender (Male)	1.329 (0.952 - 1.856)	1.041 (0.672 - 1.613)	-
Age	1.063 (1.046 - 1.080)	1.066 (1.050 - 1.083)	1.059 (1.042 - 1.077)
BMI	0.993 (0.953 - 1.034)	0.979 (0.933 - 1.026)	-
Weight	0.995 (0.989 - 1.001)	0.999 (0.992 - 1.007)	-
History of Smoking	1.956 (1.370 - 2.794)	1.929 (1.337 - 2.784)	1.884 (1.303 - 2.725)
Cholesterol	0.992 (0.986 - 0.997)	0.995 (0.989 - 1.001)	-
LDL-C	1.004 (0.999 - 1.009)	1.006 (1.001 - 1.010)	1.005 (1.001 - 1.010)
HDL-C	0.969 (0.955 - 0.984)	0.976 (0.960 - 0.992)	0.974 (0.959 - 0.989)
Hypertension	1.057 (0.741 - 1.508)	0.643 (0.435 - 0.951)	0.840 (0.583 - 1.211)*
Coronary Artery Disease	2.074 (1.497 - 2.874)	1.308 (0.878 - 1.948)	-
Diabetes	2.490 (1.797 - 3.450)	2.319 (1.653 - 3.252)	2.535 (1.773 - 3.624)
Myocardial Infarction	1.105 (0.555 - 2.200)	0.858 (0.412 - 1.788)	-
CABG	3.719 (1.624 - 4.552)	1.501 (0.847 - 2.659)	-
PCI	0.971 (0.544 - 1.733)	0.844 (0.448 - 1.591)	-
CVA	3.194 (1.281 - 3.760)	1.197 (0.673 - 2.126)	-
TIA	1.737 (0.890 - 3.350)	1.536 (0.767 - 3.077)	-
Beta-blocker	2.274 (1.524 - 3.394)	1.676 (1.109 - 2.535)	1.625 (1.064 - 2.482)
ACE-I	1.543 (1.102 - 2.162)	1.040 (0.712 - 1.519)	-
ARB	1.328 (0.906 - 1.948)	1.149 (0.760 - 1.737)	-
Omega-3	0.561 (0.382 - 0.823)	0.547 (0.369 - 0.811)	0.554 (0.373 - 0.823)
Aspirin	1.231 (0.827 - 1.830)	0.841 (0.546 - 1.295)	-
Diuretic	2.020 (1.447 - 2.820)	1.407 (0.961 - 2.059)	1.461 (1.011 - 2.112)
Ezetimibe	0.946 (0.644 - 1.390)	1.093 (0.733 - 1.628)	1.067 (0.713 - 1.598)**

ACE-I: angiotensin converting enzyme inhibitor; ARB: angiotensin II receptor blocker; BMI: body mass index; CABG: coronary artery bypass graft; CVA: cerebrovascular accident; HDL-C: high density lipoprotein cholesterol; LDL-C: low density lipoprotein cholesterol; PCI: percutaneous coronary intervention; TIA: transient ischemia attack; *Hypertension was added back into the model to control confounding; **Ezetimibe was added back into the stepwise model because it drops out. P values < 0.05 for all odds ratios by stepwise model except * and **.

## Discussion

This study adds to the growing body of evidence that ezetimibe may not provide substantial outcomes benefit in the treatment of hyperlipidemia. Our data indicate that there is no apparent mortality benefit for ezetimibe over mid-term follow up after one to three years of use.

Two trials where ezetimibe has been shown to have clinical efficacy are the SEARS and the SHARP trial. In the SEAS trial (Simvastatin and Ezetimibe in Aortic Stenosis) which compared patients on simvastatin 40 mg and ezetimibe 10 mg to those on placebo, fatal and nonfatal myocardial infarctions were significantly reduced with associated reductions in LDL-C. The SHARP trial demonstrated a 17% reduction in major atherosclerotic events. However, both these trials involve use of a statin and ezetimibe in comparison with a placebo and the observed clinical benefits could be due to the statin alone [9, 10]. In contrast our study compares the mortality benefit in patients taking statin and ezetimibe against those taking statin alone. To our knowledge ours is the first study that attempts to look at the clinical efficacy of ezetimibe in comparison to a statin alone and demonstrated no additive benefit for using ezetimibe.

Ezetimibe works by inhibiting the cholesterol absorption in the jejunum while statins work by inhibiting cholesterol synthesis by inhibiting the enzyme HMG CoA reductase [12]. Reduced hepatic synthesis of LDL-C by the statins results in up regulation of hepatic LDL receptors and consequent increase in uptake of LDL-C from the blood resulting in reduced serum LDL levels [13]. However, these effects also result in enhanced cholesterol absorption. Thus ezetimibe which acts by inhibiting cholesterol absorption in the jejunum is supposed to have a complementary mechanism of action to that of the statins and the combination has been shown to achieve LDL-C reductions that have not been possible with statin monotherapy alone. However, these reductions in LDL-C have failed to demonstrate clinical benefits as seen in the ENHANCE and the ARBITER 6 HALTS trials [5, 8]. Thus the randomized control trial (IMPROVE IT) which is testing clinical events in acute coronary syndrome patients on ezetimibe in addition to intensive statin treatment is highly anticipated.

Limitations: Though this study indicates a lack of clinical efficacy for ezetimibe, it does face several limitations. Despite the large sample size, the data only comes from one group of cardiologists at one medical center and is retrospective. Our study did not include lipid values following administration of ezetimibe. Thus the benefits of LDL reduction could not be demonstrated in our study. However the primary aim was to look at the clinical endpoint of mortality as LDL reduction by ezetimibe has already been well established.

### Conclusion

No significant mortality benefit was found with the use of ezetimibe in combination with a statin. Further randomized trials investigating the clinical efficacy of ezetimibe in the general population are needed.

## Declaration

All authors take responsibility for all aspects of the reliability and freedom from bias of the data and their discussed interpretation.
